# Radiomic Score as a Potential Imaging Biomarker for Predicting Survival in Patients With Cervical Cancer

**DOI:** 10.3389/fonc.2021.706043

**Published:** 2021-08-16

**Authors:** Handong Li, Miaochen Zhu, Lian Jian, Feng Bi, Xiaoye Zhang, Chao Fang, Ying Wang, Jing Wang, Nayiyuan Wu, Xiaoping Yu

**Affiliations:** ^1^Department of Radiology, Hunan Cancer Hospital, The Affiliated Cancer Hospital of Xiangya School of Medicine, Central South University, Changsha, China; ^2^Central Laboratory, Hunan Cancer Hospital, The Affiliated Cancer Hospital of Xiangya School of Medicine, Central South University, Changsha, China; ^3^Department of Clinical Pharmaceutical Research Institution, Hunan Cancer Hospital, Affiliated Tumor Hospital of Xiangya Medical School of Central South University, Changsha, China; ^4^Gynecological Oncology Clinical Research Center, Hunan Cancer Hospital, Affiliated Tumor Hospital of Xiangya Medical School of Central South University, Changsha, China

**Keywords:** cervical cancer, computed tomography, radiomics, nomogram, overall survival

## Abstract

**Objectives:**

Accurate prediction of prognosis will help adjust or optimize the treatment of cervical cancer and benefit the patients. We aimed to investigate the incremental value of radiomics when added to the FIGO stage in predicting overall survival (OS) in patients with cervical cancer.

**Methods:**

This retrospective study included 106 patients with cervical cancer (FIGO stage IB1–IVa) between October 2017 and May 2019. Patients were randomly divided into a training cohort (*n* = 74) and validation cohort (*n* = 32). All patients underwent contrast-enhanced computed tomography (CT) prior to treatment. The ITK-SNAP software was used to delineate the region of interest on pre-treatment standard-of-care CT scans. We extracted 792 two-dimensional radiomic features by the Analysis Kit (AK) software. Pearson correlation coefficient analysis and Relief were used to detect the most discriminatory features. The radiomic signature (i.e., Radscore) was constructed *via* Adaboost with Leave-one-out cross-validation. Prognostic models were built by Cox regression model using Akaike information criterion (AIC) as the stopping rule. A nomogram was established to individually predict the OS of patients. Patients were then stratified into high- and low-risk groups according to the Youden index. Kaplan–Meier curves were used to compare the survival difference between the high- and low-risk groups.

**Results:**

Six textural features were identified, including one gray-level co-occurrence matrix feature and five gray-level run-length matrix features. Only the FIGO stage and Radscore were independent risk factors associated with OS (*p* < 0.05). The C-index of the FIGO stage in the training and validation cohorts was 0.703 (95% CI: 0.572–0.834) and 0.700 (95% CI: 0.526–0.874), respectively. Correspondingly, the C-index of Radscore was 0.794 (95% CI: 0.707–0.880) and 0.754 (95% CI: 0.623–0.885). The incorporation of the FIGO stage and Radscore achieved better performance, with a C-index of 0.830 (95% CI: 0.738–0.922) and 0.772 (95% CI: 0.615–0.929), respectively. The nomogram based on the FIGO stage and Radscore could individually predict the OS probability with good discrimination and calibration. The high-risk patients had shorter OS compared with the low-risk patients (*p* < 0.05).

**Conclusion:**

Radiomics has the potential for noninvasive risk stratification and may improve the prediction of OS in patients with cervical cancer when added to the FIGO stage.

## Introduction

Cervical cancer is one of the fourth most common female malignancies worldwide (1). More than 80% of patients are typically diagnosed at a locally advanced stage (2). Five-year overall survival (OS) can be significantly distinct, ranging from 80% (stage IB) to 15% (stage IVa-b) (3). Despite the fact that outcomes of cervical cancer had been improved with multimodality treatment, around 30%–40% of patients still suffer from recurrence (4). Thus, it is of great significance to identify high-risk patients who may benefit from aggressive treatment.

The International Federation of Gynecology and Obstetrics (FIGO) stage has been established as the most crucial prognostic factor for cervical cancer (5). The treatment modality choice is mainly based on the FIGO stage and N staging (6). However, clinical outcomes are markedly different among patients with similar stages (6). Imaging plays an essential role in the pre-treatment evaluation of cervical cancer. However, conventional medical images only provide structural information of cancer; it fails to detect the intratumoral heterogeneity associated with treatment response and prognosis (7). Thus, the search for new non-invasive biomarkers with the potential to offer more specific tumor characterization before therapy is urgently needed, which may inform clinicians to make a more individualized treatment plan.

Statistical models, medical images, and machine learning have been widely used for outcome prediction in cervical cancer. Machine learning has merits in dealing with the complexity of high-dimensional data and discovering prognostic factors. Radiomics refers to a variety of mathematical methods such as machine learning that converts digital medical images into a huge number of minable high-dimensional features for cancer diagnosis or prediction (8, 9). Radiomic signature can be used as a surrogate biomarker for biological tumor traits such as tumor morphology and intratumor heterogeneity (10, 11). Currently, radiomics has been used to predict tumor stage, histological type, lymph node metastasis, relapse, and survival in patients with cervical cancer (12). However, the additional value of radiomics to the FIGO stage in prognostication of cervical cancer remains unclear. Thus, this study aimed to develop and validate a radiomic model for predicting survival in patients with cervical cancer.

## Materials and Methods

### Patient Cohort

This retrospective study included patients with a diagnosis of cervical cancer between October 2017 and May 2019 at our institution. Inclusion criteria were (1) patients with histologically confirmed cervical cancer, (2) patients with tumor staged IB1-IVa (FIGO 2009 definition), (3) patients who were not previously treated with any anti-cancer treatment, and (4) patients who underwent a pre-treatment contrast-enhanced computed tomography (CT) scan. Exclusion criteria were (1) patients with a history of previous chemotherapy or radiotherapy, (2) patients with a diagnosis of other cancers, or (3) patients with distant metastatic disease (para-aortic nodes involvement was not included). Institutional ethics review board approval was acquired for this study and written informed consent was waived. Finally, a total of 106 patients (mean age, 63.8 years) were included in this study. Eligible patients for the radiomic study were randomly divided into a training cohort (*n* = 74) and a validation cohort (*n* = 32). The clinical information of patients was collected from electronic medical records, including age, FIGO stage, histological type, differentiation, lymph node metastasis (LNM), and treatment regimens. Of note, a lymph node with a short-axis diameter larger than 10 mm was considered to be metastasis (13).

### Treatment Characteristics and Follow-Up

All patients were treated with image-guided external beam radiotherapy (EBRT) and brachytherapy (BT), to a total dose of 85–90 Gy (EQD2, equivalent dose in 2 Gy single dose fractions). EBRT was delivered in 1.8–2.0 Gy/fraction, to a range of 45–50 Gy, using a 3D conformal technique. BT boost was volumetrically planned and delivered as weekly high-dose-rate fractions of 8 Gy EQD2 each after 15 times of EBRT. The external irradiation was not performed on the day of intracavitary and interstitial after loading BT. The radiation volumes covered the pelvic cavity and, if clinically indicated, the para-aortic and/or inguinal nodal regions. CT-positive lymph nodes were simultaneously boosted to a total dose of 55–60 Gy. Concurrent weekly chemotherapy with cisplatin (40 mg/m^2^) was delivered for 4–6 weeks or carboplatin (AUC = 2) when feasible. The endpoint OS was defined as the interval from the date of treatment to death from any cause. The patients were followed up until November 19, 2020.

### CT Image Acquisitions

CT scans were performed on a 64-row CT scanner (Somatom definition AS large-aperture, Siemens Healthcare) using the following parameters: 120-kVp tube voltage, 252-mAs tube current, a field of view (FOV) of 384 × 384 mm^2^, a width of detector of 40 mm, a beam pitch of 0.6, a gantry rotation time of 0.5 s, and a slice thickness of 5 mm. Iohexol (350 mg I/ml) was administrated with a rate of 2.5–3.0 ml/s through the elbow vein by a high-pressure injector. Enhanced CT images were obtained at 30 s after injection.

### Radiomic Analysis

**Figure 1:**
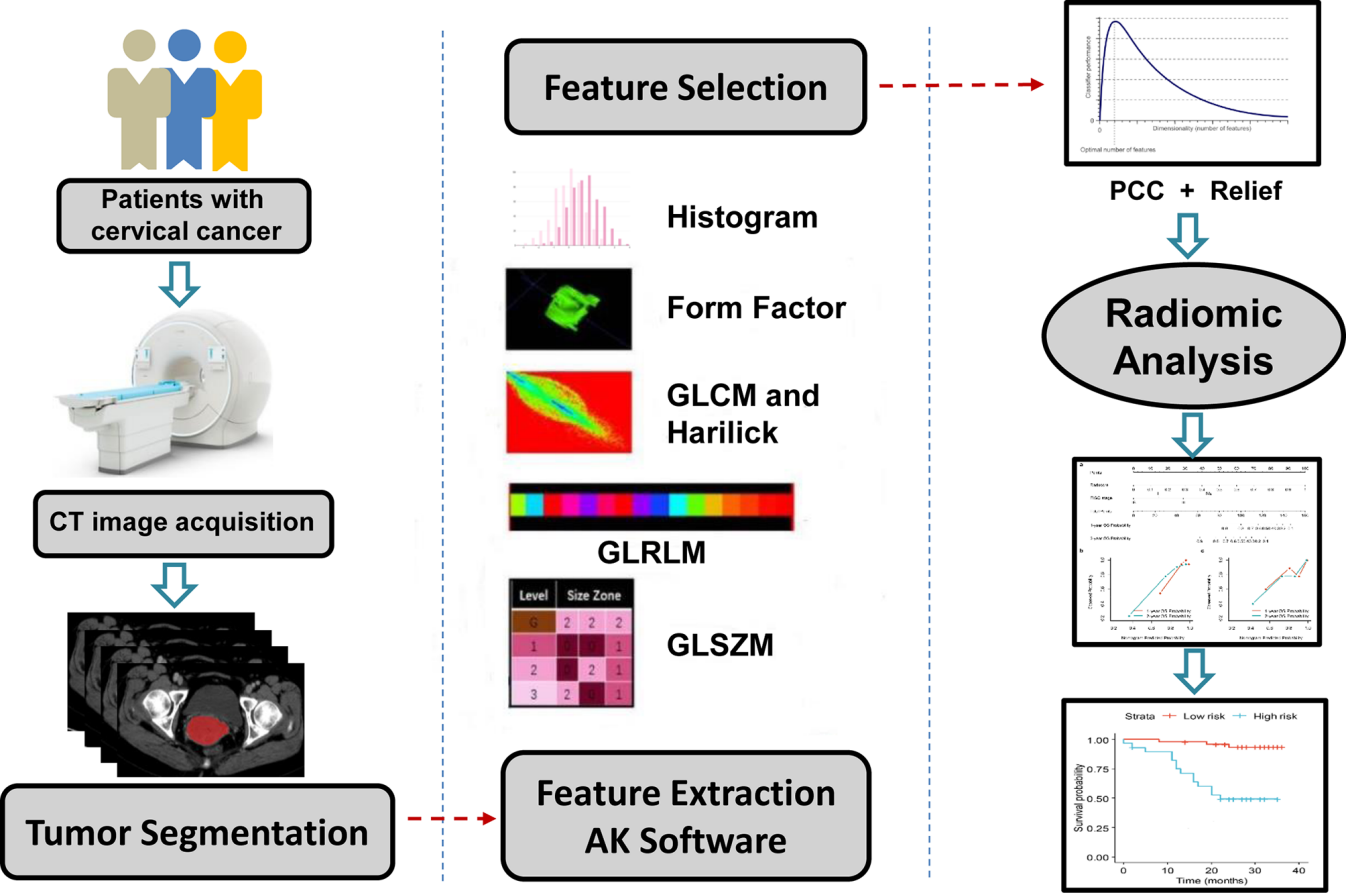
Schematic diagram exhibition of the radiomic workflow. A radiomic study design and workflow mainly include (I) Image segmentation, (II) Radiomic feature extraction, (III) Dimension reduction and feature selection, (IV) Statistics analysis and model building. CT, computed tomography; GLCM, gray-level co-occurrence matrix; GLZSM, gray-level size zone matrix; GLRLM, gray-level run-length matrix; PCC, Pearson correction coefficient.

Our radiomic workflow is illustrated in **Figure 1**.

#### Image Segmentation

**Figure 2:**
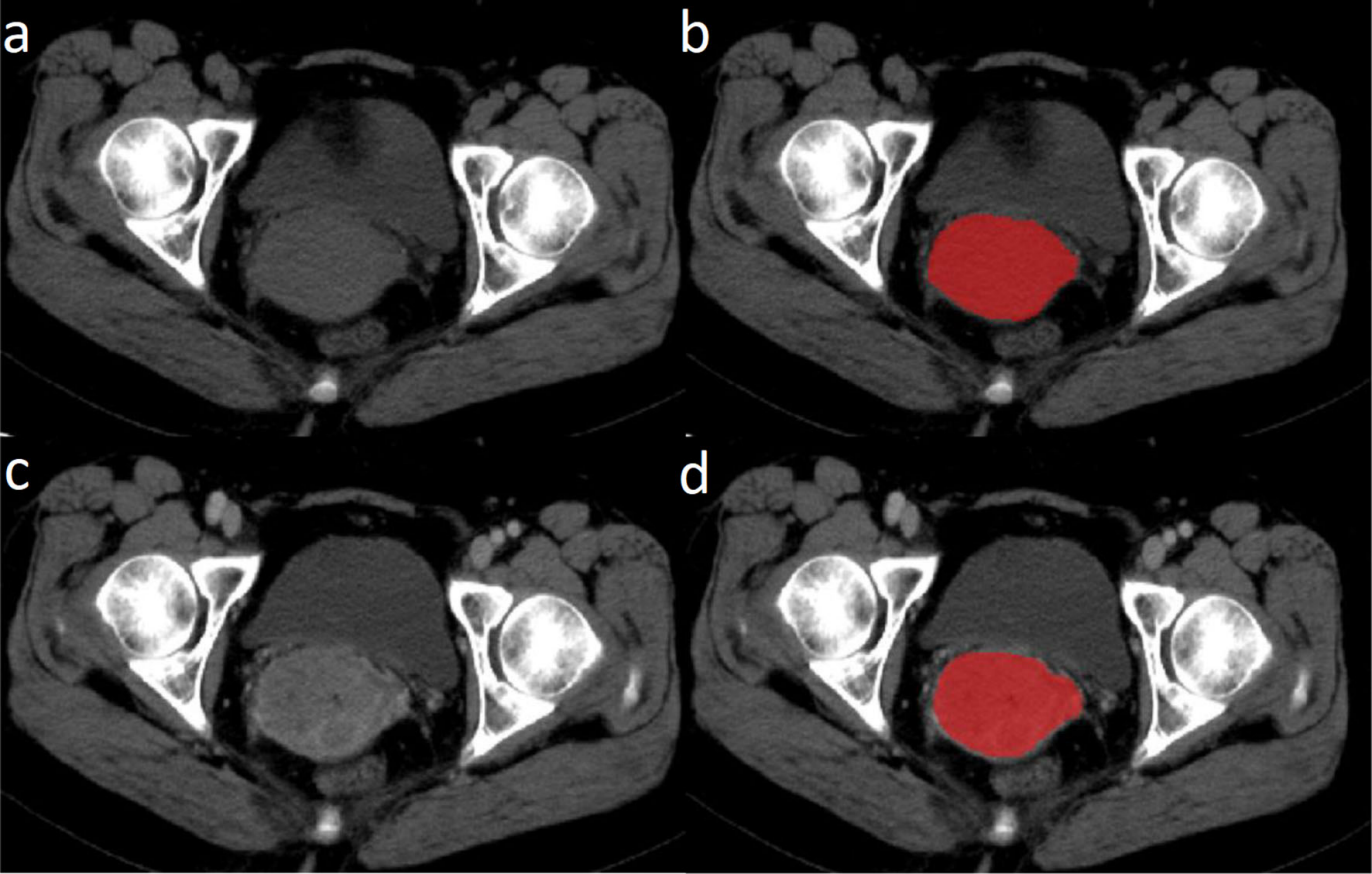
Illustration of tumor segmentation on the maximum level of tumor. **(A)** Raw pre-contrast image; **(B)** pre-contrast image after delineation; **(C)** raw post-contrast image; and **(D)** post-contrast image after delineation.

We used an open-source ITK-SNAP software (version 3.6.0, www.itksnap.org) for manual segmentation on CT images (**Figure 2**). Tumor lesion segmentation was performed on the maximum level of tumor by a radiologist (with 6-year experience) and subsequently reviewed by a board-certified radiologist (>10 years experience).

#### Feature Extraction

The radiomic features were automatically calculated by AK software (Artificial Intelligence Kit, GE Life Sciences, AA R&D team, Shanghai, China), which comply with the standards set by the Image Biomarker Standardization Initiative. In total, 792 radiomic features were extracted from pre-contrast and post-contrast CT images, including (1) histogram features, such as energy, entropy, uniformity, skewness, and kurtosis; (2) form factor features, such as sphericity, surface area, compactness, surface volume ratio, maximum 3D diameter, spherical disproportion, volume CC, and volume MM; and (3) texture features including gray-level co-occurrence matrix (GLCM), gray-level size zone matrix (GLZSM), gray-level run-length matrix (GLRLM), and Haralick parameters. The offsets of GLCM and GLRLM were 1, 4, and 7.

#### Feature Pre-Processing and Selection

Feature pre-processing was done in two steps: step 1—outliers and null values were replaced by mean values, and step 2—values standardization was carried out to eliminate the influence of the dimension (14). Feature selection is a critically important step for better generalization of models because high-dimensional data usually comprise a large number of irrelevant, redundant, and noisy features, which may result in the curse of dimensionality and model overfitting (15). In terms of feature selection, Pearson correction coefficient (PCC) analysis was used to assess the correlation between feature pairs, and one feature was randomly excluded from each pair with a correlation coefficient >0.9. After this process, the dimension of the variable space was reduced, and each variable was independent of each other. Then, we used Relief, a feature weighting algorithm, to detect the most discriminatory features. Finally, radiomic signature (i.e., Radscore) based on the selected features was constructed *via* Adaboost with Leave-one-out cross-validation.

### Prognostic Model Construction

Variables with missing data of more than 20% were excluded for analysis. The significant variables were identified by the univariate and multivariate Cox regression model. The variables with *p* < 0.10 in the univariate analysis were entered into multivariate analysis to obtain independent risk factors for OS (*p* < 0.05). The prognostic models were constructed by Cox proportional hazard model, using the Akaike information criterion (AIC) as the stopping rule. For convenient use by clinicians, we built a nomogram to individually predict the OS of patients based on the optimal prognostic model. The calibration curves reflecting the goodness of fit of the nomograms generated were assessed by plotting the predicted probabilities against the observed event proportions. The receiver operating characteristic (ROC) curve was used to determine cutoff values of models according to the Youden index to generate Kaplan–Meier curves for OS in the training and validation cohorts. The Log-rank test was used for comparisons in the Kaplan–Meier curves.

### Statistical Analysis

The clinicopathologic characteristics were assessed by applying two-sample *t*-test, chi-squared test, or Mann–Whitney *U*-test in the training and validation cohorts, where appropriate. The discrimination of each model was quantified by the C-index and 95% confidence interval (CI). The models were subjected to bootstrapping validation (1000 bootstrap resamples) to obtain a relatively corrected C-index. All statistical analyses were performed using 3.6.0 R software (http://www.Rproject.org). A two-tailed *p* < 0.05 was considered statistically significant.

## Results

### The Patient Cohort of the Radiomic Study

There were no significant differences in the clinicopathologic characteristics between the training and validation cohorts (all *P* values >0.05) (**Table 1**).

**Table 1:** Patients’ characteristics.

Characteristics	Training cohort (*n* = 74)	Validation cohort (*n* = 32)	*p*-value
**Age (years)**	59.0 ± 8.3	60.3 ± 9.7	0.495
**FIGO stage**			0.427
IB	1 (1.4)	2 (6.3)	
II	41 (55.4)	20 (62.5)	
III	28 (37.8)	9 (28.1)	
IVa	4 (5.4)	1 (3.1)	
**Histological type**			0.866
Squamous	68 (91.9)	30 (93.8)	
Adenocarcinoma	4 (5.4)	1 (3.1)	
Adenosquamous carcinoma	2 (2.7)	1 (3.1)	
**Lymph node involvement on CT**			0.206
Uninvolved	29 (39.2)	17 (53.1)	
Involved	45 (60.8)	15 (46.9)	
**Differentiation**			0.999
Poor	3 (4.1)	1 (3.1)	
Poor-moderate	12 (16.2)	5 (15.6)	
Moderate	41 (55.4)	18 (56.3)	
Well-moderate	4 (5.4)	2 (6.3)	
Unknown	14 (18.9)	6 (18.8)	
**Concurrent chemotherapy**			0.602
With	60 (81.1)	24 (75.0)	
Without	14 (18.9)	8 (25.0)	
Median OS (months)	27.0	28.0	0.756

Data were expressed as number (percentage) or mean (standard deviation). FIGO, International Federation of Gynecology and Obstetrics; OS, overall survival.

### Feature Selection and Radscore Construction

A total of 428 radiomic features were retained after PCC analysis; only six texture features were then selected by Relief, including one GLCM feature (pre_*GLCMEntropy_AllDirection_offset7_SD*) and five GLRLM features (post_*LongRunEmphasis_angle135_offset1.1*, pre_*LongRunEmphasis_AllDirection_offset1_SD.1*, pre_*LongRunLowGreyLevelEmphasis_AllDirection_offset1*, post_*LongRunEmphasis_angle45_offset1*, and post_*LongRunHighGreyLevelEmphasis_AllDirection_offset4_SD.1*). Pre_ and post_ denote the pre-contrast and post-contrast features, respectively. The histogram and form factor features were omitted. The Radscore was built by the Adaboost classifier based on the six textural features.

### Independent Indicators of OS

Multivariate Cox regression analysis showed that among clinicopathologic characteristics, only the FIGO stage was an independent factor of OS (*p* < 0.002). The hazard ratio (HR) for the FIGO stage was 3.24 (95% CI: 1.55–6.76). When adding the Radscore to the aforementioned features, only Radscore was an independent indicator of OS (*p* = 0.002). The HRs for the FIGO stage and Radscore were 2.07 (95% CI: 0.89–4.83) and 153.0 (95% CI: 6.29–3721.16), respectively.

### Performance of Prognostic Models

**Figure 3:**
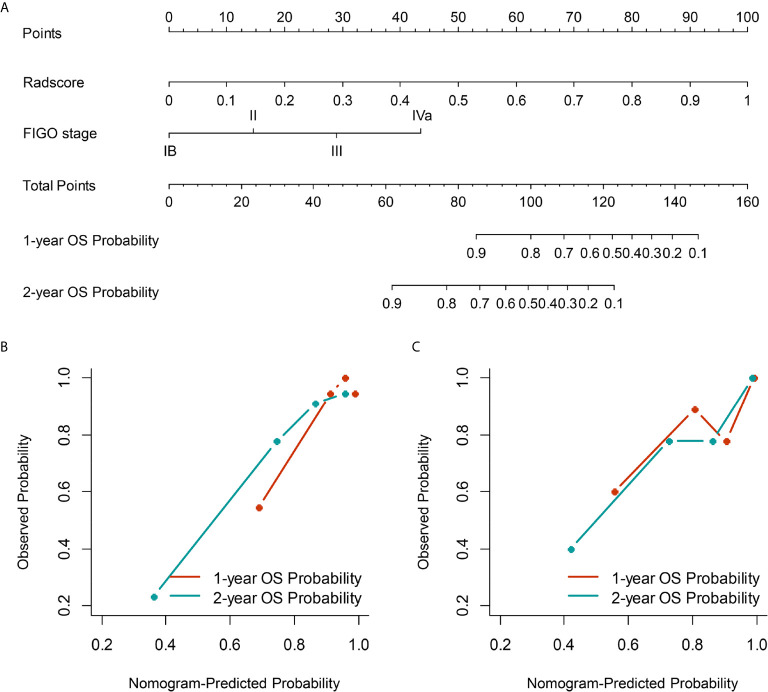
The nomogram **(A)** based on the FIGO stage and Radscore was used to estimate OS individually, along with the assessment of the model calibration. Calibration curves for the nomogram to the 1-year and 2-year OS rate in the training **(B)** and validation cohorts **(C)**. FIGO, International Federation of Gynecology and Obstetrics; OS, overall survival.

The C-index of the FIGO stage in the training and validation cohorts was 0.703 (95% CI: 0.572–0.834) and 0.700 (95% CI: 0.526–0.874), respectively (**Table 2**). The C-index of Radscore was 0.794 (95% CI: 0.707–0.880) and 0.754 (95% CI: 0.623–0.885), respectively (**Table 2**). The incorporation of the FIGO stage and Radscore achieved better performance, with a C-index of 0.830 (95% CI: 0.738–0.922) and 0.772 (95% CI: 0.615–0.929), respectively (**Table 2**). The nomogram based on the FIGO stage and Radscore could individually predict the OS probability (**Figure 3**). Calibration curves demonstrated good agreement between the predicted probability and observed probability (**Figure 3**).

**Table 2:** The performance of FIGO stage, Radscore, and the combined model for OS evaluation in patients with cervical cancer.

Models	AIC	C-index (95% CI)	
		Training cohort	Validation cohort
FIGO stage	132.9	0.703 (0.572–0.834)	0.700 (0.526–0.874)
Radscore	121.4	0.794 (0.707–0.880)	0.754 (0.623–0.885)
Combined model	120.5	0.830 (0.738–0.922)	0.772 (0.615–0.929)

FIGO, International Federation of Gynecology and Obstetrics; AIC, Akaike information criterion; CI, confidence interval; OS, overall survival.

### Kaplan–Meier Survival Analysis

**Figure 4:**
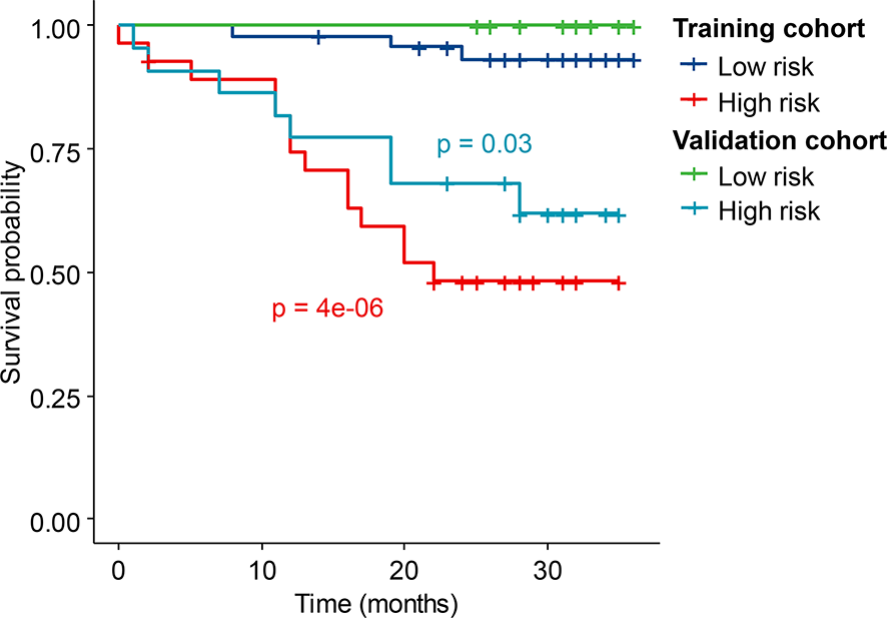
Kaplan–Meier curves of the high- and low-risk patients stratified by the combined model in the training cohort and validation cohort.

Kaplan–Meier curves demonstrate that by using a cutoff value of 0.59, Radscore alone could stratify patients into the low- and high-risk groups, with significant OS differences. The high-risk patients had significantly shorter OS than the low-risk patients (training cohort: *p* < 0.0001; validation cohort: *p* = 0.03). The combined FIGO stage and Radscore could achieve better risk stratification using a cutoff value of 0.57 (training cohort: *p* < 0.0001, validation cohort: *p* = 0.02) (**Figure 4**).

## Discussion

In this radiomic study, we demonstrated that capturing intratumoral radiomics from pretherapy CT images could significantly improve the performance of the FIGO stage for OS evaluation. The combined model yielded the optimal performance for OS prediction in patients with cervical cancer. We established a radiomic nomogram derived from a combined model to individually estimate the OS of patients. The nomogram could successfully stratify these patients into high-risk and low-risk subgroups, and these two subgroups had different OS in both training and validation cohorts.

Prognosis evaluation in advance will greatly improve the treatment outcome in patients with cervical cancer. The most relevant tumor-related prognostic factors for locally advanced cervical cancer are tumor size at diagnosis, FIGO stage, and LNM (6, 16). However, our work showed that only the FIGO stage was an independent risk factor of OS in stage IB1–IVa cervical cancer. FIGO stage is the current standard staging system for cervical cancer based on findings from physical inspection and imaging examinations (17). The limitations of the FIGO stage are that it depends on many examinations and its classification is not objective enough (18). Therefore, an objective and efficient evaluation method that complements cervical cancer staging is of clinical significance.

The current radiomic workflow consists of adding quantitative data to visual analysis rather than replacing it entirely (19). In the field of oncology, translational approaches are increasingly explored, for instance, Radiogenomics. Such studies usually need “big data” approaches and collaborative research (20). In this work, we proposed a new approach, translational but able to be quickly implemented for clinicians. To the best of our knowledge, this is the first study to integrate accessible FIGO stage and radiomic features extracted from pre-treatment CT images to predict the survival of cervical cancer. Radscore may be a more powerful predictor compared with the FIGO stage. With the help of Radscore, the prognostic value of the FIGO stage was significantly improved, leading to better risk stratification of patients. This score may be used for adaptive strategies to improve patient outcomes.

Radiomics has been involved in many aspects of cervical cancer, including prediction of tumor staging (21, 22), histological grading (23, 24), lymphovascular space invasion (LVI) (25), LNM (26–36), treatment response (37–42), and outcome (43–48) based on multimodal imaging tools (e.g., CT, MRI, and PET/CT). In clinical practice, the issues of most concern may be the evaluation of LNM, treatment response, and survival prediction. Hence, most radiomic studies focused on the evaluation of these in a non-invasive way. LNM is a key prognostic factor that affects the treatment decision and survival in cervical cancer. Song et al. combined the Radscore and morphological features of lymph nodes to assess the LNM in cervical cancer patients (26). Some studies developed radiomic models based on Radscore and clinical factors (e.g., MRI-reported lymph node status and FIGO stage) for predicting LNM in cervical cancer (27–36). Treatment response to neoadjuvant or concurrent chemotherapy may have a significant impact on patient management by identifying tailored approaches for patient subgroups to achieve a better clinical outcome. All relevant studies showed the additional value of radiomics to clinical factors and its potential to screen patients who are sensitive to chemotherapy (37–42). Survival evaluation is an eternal proposition for the field of oncology, which may benefit treatment strategies and follow-up plans. Ferreira et al. tested the feasibility of PET radiomic features combined with clinical information in predicting disease-free survival (DFS) in patients with cervical cancer (43). Fang et al. used MRI-based Radscore, LNM, and LVI to predict the DFS of early-stage cervical cancer (45). Lucia et al. found that the combination of PET/CT and MRI could result in more favorable survival prediction (47). Another study suggested that Radscore could enhance the prediction efficiency of conventional PET parameters in cervical cancer (48). In this current study, the results indicated that Radscore was an independent risk factor strongly associated with OS of cervical cancer and only texture features contributed to the survival prediction in cervical cancer. The HR of the Radscore was significantly higher than that of the FIGO stage, suggesting that the Radscore may provide more prognostic information than the FIGO stage in predicting the OS.

This study also had some limitations. First, selection bias was inevitable because of a retrospective study. Second, the sample size was small for a radiomic study. However, we performed a bootstrap to obtain corrected results. Third, the overall treatment times were not readily available, which is a well-recognized factor in cervical cancer outcomes. Fourth, this study had no local/regional failures, recognizing the short follow-up time. The additional evaluation of local control could be more clinically significant. Fifth, our radiomic features were extracted from CT images; those features from MRI or PET/CT images may provide different information about intratumoral heterogeneity. Sixth, the image analysis was based on the maximum level of the tumor instead of the whole tumor. However, some previous studies (45, 49) showed that the predictive performance of features extracted from the maximum level of the tumor was higher than that of those features extracted from the whole tumor. Two-dimensional features may increase the robustness of features compared with three-dimensional features. Seventh, the biological interpretation of radiomics remains an open question warranting further investigation (50). Finally, this is a single-institution study that needs external validation of the findings.

In conclusion, this present study provided a combined model, incorporating the FIGO stage and a Radscore derived from CT-based textural features, with favorable performance, and the study developed a noninvasive radiomic nomogram based on the results of the combined model for the pretherapy and personalized estimation of OS in patients with cervical cancer. In addition, the combined model serves as a collection of potential biomarkers and perfectly stratifies these patients into high-risk and low-risk subgroups. The identification of high-risk patients at diagnosis can allow tailored treatments involving higher doses of radiation boost, consolidation chemotherapy, and/or adjuvant hysterectomy, when indicated, and should be confirmed in external cohorts and prospective studies.

## Data Availability Statement

The original contributions presented in the study are included in the article/supplementary material. Further inquiries can be directed to the corresponding authors.

## Ethics Statement

The studies involving human participants were reviewed and approved by Hunan Cancer Hospital, The Affiliated Cancer Hospital of Xiangya School of Medicine, Central South University. Written informed consent was waived.

## Author Contributions

HL, MZ, and LJ designed the study. HL, MZ, and LJ contributed to the conception of the study and completed the manuscript together. JL, FB, and XZ contributed significantly to statistical analysis. YW and JW completed the following-up information. LJ, NW, and XY drafted the manuscript. All authors contributed to the article and approved the submitted version.

## Conflict of Interest

The authors declare that the research was conducted in the absence of any commercial or financial relationships that could be construed as a potential conflict of interest.

## Publisher’s Note

All claims expressed in this article are solely those of the authors and do not necessarily represent those of their affiliated organizations, or those of the publisher, the editors and the reviewers. Any product that may be evaluated in this article, or claim that may be made by its manufacturer, is not guaranteed or endorsed by the publisher.
